# Minimally Invasive Surfactant Administration for the Treatment of Neonatal Respiratory Distress Syndrome: A Multicenter Randomized Study in China

**DOI:** 10.3389/fped.2020.00182

**Published:** 2020-05-07

**Authors:** Tongyan Han, Huiqiang Liu, Hui Zhang, Ming Guo, Xuefeng Zhang, Yang Duan, Fuqiang Sun, Xinjian Liu, Xiang Zhang, Mingtao Zhang, Fang Liu, Lisha Bao, Min Xiao, Weili Liu, Rui Jiang, Jun Zheng, Xiuying Tian, Qi Gao, Wanxian Zhang, Wei Guo, Ling Li, Xiaomei Tong

**Affiliations:** ^1^Department of Pediatrics, Peking University Third Hospital, Beijing, China; ^2^Department of Neonatology, Fifth Medical Center, General Hospital of the Chinese People's Liberation Army, Beijing, China; ^3^Department of Neonatology, Second Hospital of Tianjin Medical University, Tianjin, China; ^4^Department of Neonatology, Hebei PetroChina Central Hospital, Langfang, China; ^5^Department of Neonatology, Bethune International Peace Hospital, Shijiazhuang, China; ^6^Department of Neonatology, Cangzhou Central Hospital of Hebei Province, Cangzhou, China; ^7^Department of Neonatology, Tianjin Central Hospital of Obstetrics and Gynecology, Tianjing, China; ^8^Department of Neonatology, Xingtai People's Hospital, Xingtai, China

**Keywords:** minimally invasive surfactant administration, neonatal respiratory distress syndrome, bronchopulmonary dysplasia, patent ductus arteriosus, extremely low birth weight infants, preterm infants

## Abstract

**Background/Aims:** Nasal continuous positive airway pressure (nCPAP) was recommended as the initial respiratory support for spontaneous breathing in infants with very low birth weight and neonatal respiratory distress syndrome (NRDS). Less invasive surfactant administration (LISA) and minimally invasive surfactant therapy (MIST) have been reported to reduce the incidence of bronchopulmonary dysplasia (BPD). This study aimed to explore the applicability of minimally invasive surfactant administration (MISA) in China.

**Materials and Methods**: MISA was a randomized controlled study conducted at eight level III neonatal intensive care units (NICUs) in China. Spontaneously breathing infants born at 25+0 to 31+6 weeks' gestation who progressively developed respiratory distress during the first 6 h after birth were randomly assigned to receive MISA or endotracheal intubation surfactant administration (EISA). The primary outcome was the difference in the morbidity of BPD between two groups of infants with MISA and EISA at 36 weeks corrected gestational age.

**Results**: Demographic and clinical characteristics of the 151 infants in the MISA group were similar to the 147 infants in the EISA group. The comparison showed no clear benefits in the MISA group in the incidence of BPD, while infants from the EISA group had higher rates of patent ductus arteriosus (PDA) (60.5 vs. 41.1%, *p* = 0.001). The duration of surfactant infusion and the total time of surfactant administration in the MISA group were significantly longer than in the EISA group. A slightly increased heart rate was noted 1 h post surfactant administration in the EISA group. In subgroup analysis, the comparison of 51 smaller (<30 weeks) preterm infants, named MISAs (*n* = 31) and EISAs (*n* = 20), showed a significant reduction of BPD (29.0 vs. 70.0%, *p* = 0.004) and PDA (29.0 vs. 65.0%, *p* = 0.011). In the subgroup analysis of blood gas, arterial oxygen saturation (SaO_2_) value at 1 and 12 h and partial pressure of arterial oxygen (PaO_2_) at 12 h were all higher in the EISA group compared to the MISA group.

**Conclusion**: MISA had no clear benefit on the incidence of BPD, but it was related to a reduction in PDA. It is an appropriate therapy for spontaneous breathing in infants with extremely low birth weight and NRDS.

## Introduction

Pulmonary surfactant is key to normal alveolar expansion. It is secreted by the type II pneumocytes to decrease surface tension, leading to increased compliance and decreased atelectasis (1). Neonatal respiratory distress syndrome (NRDS) is a disorder of surfactant deficiency, especially preterm infants <32 weeks old with very low birth weight whose type II pneumocytes are too immature to produce sufficient surfactant to support normal alveolar expansion (2). Treatment includes respiratory support and exogenous surfactant. Fujiwara et al. introduced surfactant treatment into neonatology in 1980 (3), which significantly reduced the need for invasive positive pressure ventilation of infants with NRDS. Traditionally, the surfactant is administered to intubated RDS infants by intratracheal bolus on positive pressure ventilation as early as possible (4). Victorin LH, a Swedish neonatologist, was the first to use an INSURE (INSURE = INtubation SURfactant Extubation) approach in the Arab countries (5). INSURE reduces the duration of positive pressure ventilation and the risk of ventilator-induced lung injury (VILI), but still needs intubation, which means a slight damage to the endotracheal epithelium mucosae of the immature airway.

In spite of the optimal approach for the initial respiratory support of infants with extremely low birth weight (ELBW) and very low birth weight (VLBW) being uncertain, stabilization by nasal continuous positive airway pressure (nCPAP) rather than immediate intubation in delivery room is advocated for them who are breathing spontaneously (6). Even brief exposures to tracheal intubation and positive pressure ventilation can increase the risk of lung injury. Application of non-invasive respiratory support, and especially nCPAP, has become a popular strategy.

Though many ELBW/VLBW infants with surfactant deficiency disorder completely recover after numerous treatments in NICU, a subset of patients tends to develop bronchopulmonary dysplasia (BPD) (7). Northway and colleagues were the first who described this condition in 1967 (8). Nowadays, BPD primarily affects ELBW/VLBW infants who are <32 weeks' gestational age and in the canalicular and saccular stages of lung development (9). Because endotracheal intubation and brief positive pressure ventilation can be harmful to the endotracheal epithelium mucosae and immature lung, methods that would allow for less invasive surfactant administration (LISA) have been investigated. LISA that has been applied in European countries (10) and the minimally invasive surfactant therapy (MIST) are among these methods (11, 12). These were derived from two methods: the Hobart method and the Cologne method. The Hobart method, which was first described by Dargaville and coworkers, instilled surfactant by semi-rigid vascular catheter with direct laryngoscopy (12). The Cologne method put the tip of a gastric catheter through the vocal cords with the aid of the Magill's forceps (10). Infants are continued on nCPAP or intermittent nCPAP throughout the procedure.

The objective of this study was to assess whether minimally invasive surfactant administration (MISA) is applicable in preterm infants with a gestational age less than 32 weeks in China. We hypothesized that MISA decreases incidence of BPD at 36 weeks corrected gestational age compared with invasive endotracheal intubation surfactant administration (EISA).

## Materials and Methods

### Study Design and Patients

This MISA study was a multicenter, randomized controlled study completed at eight level III neonatal intensive care units (NICUs) in Beijing, Tianjin, and Hebei province, China, between July 1, 2017, and December 31, 2018. Each institution's Ethics Review Board approved the study. MISA is registered with ClinicalTrials.gov, NCT04077333.

A written informed consent for participation was obtained from a parent during the prenatal high-risk consultation before delivery or as quickly as possible after admission to the NICU (up to 120 min post-admission). The gestational age was estimated based on the Ballard score assessment postnatally (13). Inclusion criteria were as follows: (1) infants with gestational age between less than 31 weeks and 6 days were eligible; (2) infants with respiratory distress using NCPAP as ventilation support; (3) infants who had signs of respiratory distress (respiratory rate >60/min, with retractions, nasal flaring, grunting, or cyanosis; fraction of inspired oxygen [FiO_2_] >0.4 for transcutaneous oxygen saturation [SpO_2_] >85%) that had progressively developed, and finally needed surfactant administration within 6 h of life. Exclusion criteria were as follows: (1) infants who were intubated in the delivery room or before the surfactant administration; (2) infants with major congenital malformations affecting respiratory function; (3) infants who died or were transferred to other hospitals for surgery or with uncompleted data; (4) infants who were enrolled in other interventional studies; (5) for the MISA group, repeated surfactant doses by intubation and positive pressure ventilation support during the first 72 h were excluded.

### Randomization

Eligible infants were randomly assigned in a 1:1 ratio to receive surfactant via MISA during nCPAP (intervention group) or via EISA during positive pressure ventilation (control group). Sequentially numbered, opaque, and sealed envelopes were used to complete the group assignment. Multiple-birth infants were allocated to the same group.

### Blinding

The assigned treatment was not blinded, as the mode of respiratory management was apparent to clinicians and nurses in the NICU. All enrolled infants were given routine clinical interventions according to established guidelines or protocols. Furthermore, the primary outcome and the secondary outcomes were based on objective sets of criteria applied to both groups.

### Study Intervention

**Figure 1:**
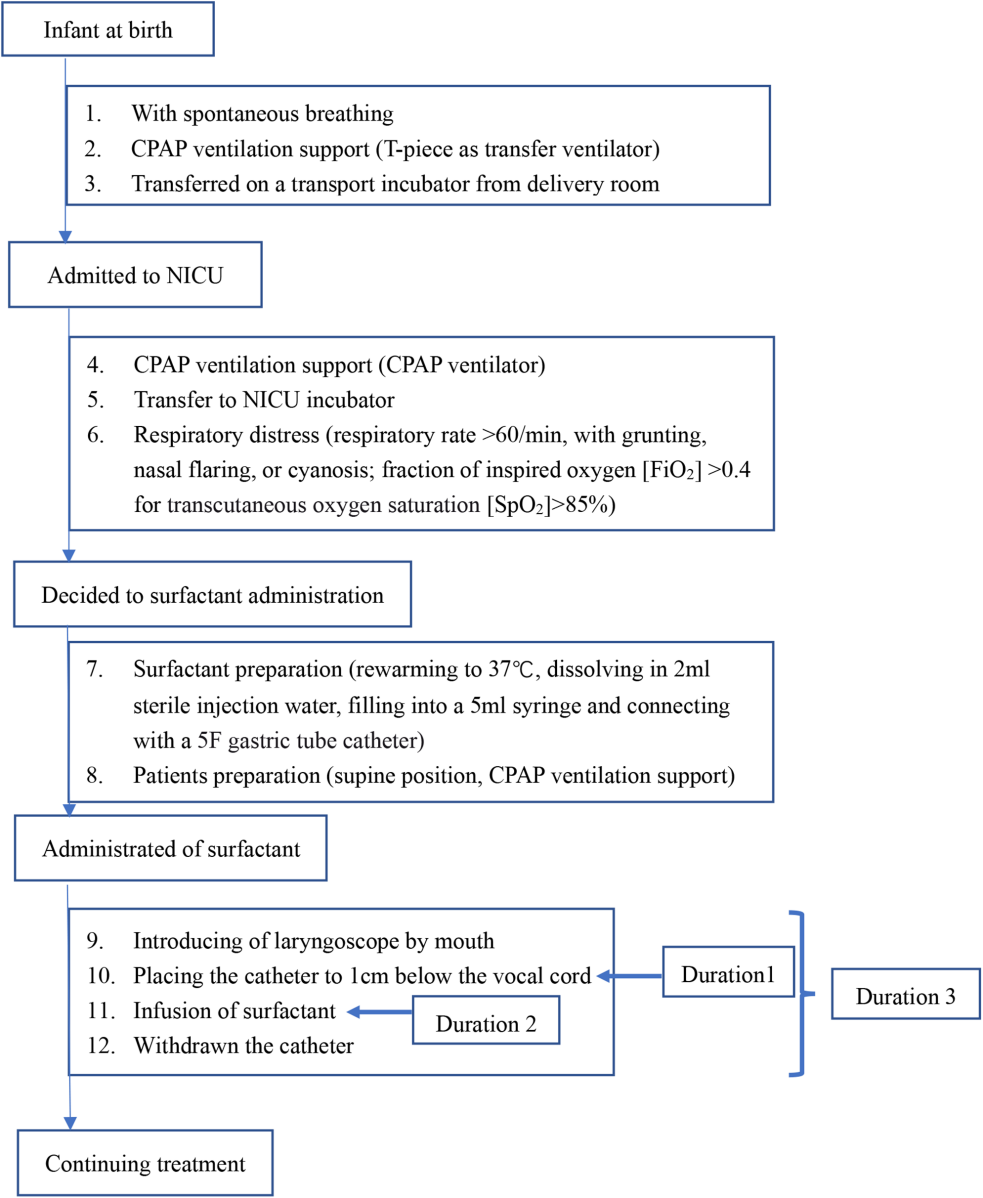
Flowchart of the administration of surfactant.

For infants in the MISA group, the intervention process was performed according to the flowchart shown in Figure 1. Once the decision regarding the surfactant administration was made, the process was initiated. A 5F end hole gastric tube catheter was used. A 5-ml syringe was prefilled at 70–100 mg/kg of body weight of the calf pulmonary surfactant preparation. This syringe was connected with the gastric tube. The tube was grasped by a 10-cm ophthalmic forceps near the tip. When the infant was breathing via nCPAP, a laryngoscope was gently introduced to provide a glottal view. The tip of the tube was positioned up to 1.0 cm below the vocal cords. The tube was tightly fixed in this position by the thumb and forefinger of the clinician. The laryngoscope and the ophthalmic forceps were removed, and the infant's mouth was closed. The surfactant was instilled by a nurse over 60 to 300 s by mini-boluses. In the case of transient bradycardia, gentle massage of the infant's back skin was performed by another nurse, until recovery. The tube was immediately removed. Sedation and analgesia were not used. During surfactant administration, nCPAP therapy was continued. During the first 72 h after birth, the nCPAP level was kept within a range of 6 and 8 cmH_2_O. The nCPAP level was titrated to 5 to 6 cmH_2_O to achieve the lowest FiO_2_ level after 72 h.

For infants in the EISA group, once respiratory distress progressively developed and they were diagnosed as nCPAP failure, they were intubated and received positive pressure ventilation support. The surfactant was administered through an endotracheal tube. Sedation and analgesia were not used. Clinicians in NICUs were advised to use INSURE method. Positive pressure ventilation support was performed following predefined standards. Investigators were encouraged to wean the infant from positive pressure ventilation as soon as possible. Extubation criteria were established as FiO_2_ <0.3 and mean airway pressure (MAP) of <8 cm H_2_O.

Infants in both groups received calf pulmonary surfactant preparation (Calf pulmonary surfactant, Beijing Double-Crane Pharmaceuticals Co. Ltd.), at doses of 70–100 mg surfactant/kg of body weight. When the FiO_2_ level exceeded 0.4, with progressively developed respiratory distress, repeated doses of surfactant were allowed in both groups. For the MISA group, infants with a second dose of surfactant by MISA were included. However, if the infants needed FiO_2_ > 0.40 lasting more than 2 h during nCPAP after the first dose of surfactant and had intubated ventilation support during the first 72 h after birth, the data of the patients would be excluded from the final analysis. All infants who were spontaneously breathing and those for whom the extubation was planned received caffeine. All other clinical interventions were applied according to established standards/protocols.

### Primary and Secondary Outcomes

The primary outcome was the difference in the morbidity of BPD between MISA and EISA groups of infants at 36 weeks corrected gestational age. Infants who were supported by mechanical ventilation or nCPAP or those with a fraction of inspired oxygen (FiO_2_) exceeding 0.30 were diagnosed as having BPD, which was defined by the National Institute of Child Health and Human Development (NICHD) Neonatal Research Network (14). If the infants were discharged before 36 weeks, they were classified according to their oxygen status at discharge.

An additional pre-specified secondary outcome was the incidence of significant complications. The infants with presence of clinical signs (respiratory rate >60/min with retractions, nasal flaring, grunting, or cyanosis) who progressively developed respiratory distress and needed supplemental oxygen ([FiO_2_] >0.4 for [SpO_2_] >85%) received an RDS diagnosis following chest X-ray confirmation. In some cases, supplemental oxygen was necessary for progressively developed respiratory distress ([FiO_2_] >0.4), as well as surfactant administration before chest X-ray (6). If the chest X-ray could not confirm RDS, the infant was diagnosed as no RDS. The neonatal pneumonia was diagnosed on a combination of clinical signs, physical examination findings, and X-ray evidences from each NICU. The subtype of neonatal pneumonia included congenital pneumonia, early-onset pneumonia, and late-onset pneumonia (including ventilator-associated pneumonia, VAP) (15). Neonatal sepsis included clinical sepsis and blood culture-confirmed sepsis. Clinical sepsis was defined as clinical signs without proof of causative agent. Blood culture-confirmed sepsis was defined as clinical sepsis with proof of causative agent in the blood culture. Patent ductus arteriosus (PDA) was based on clinical signs and echocardiographic confirmation. Echocardiography was performed during screening to identify malformations, to diagnose hemodynamically significant PDA (hsPDA), and during the treatment in oral ibuprofen to monitor the closure of PDA and to estimate measurements, such as internal diameter of the duct size, pulmonary artery systolic pressure, the left ventricular ejection fraction, and so on. Surgical ligation was considered when two courses of oral ibuprofen failed to close the hsPDA (16). White matter injury and intraventricular hemorrhage (IVH) were seen on cranial ultrasound and IVH was graded as grades 1–2 and grades 3–4 (17). The diagnosis and staging of retinopathy of prematurity (ROP) were based on retinal examination, both in the NICU and post-discharge. Severe ROP was defined as stages 3–5 (18). The diagnosis of necrotizing enterocolitis (NEC) was based on more than one clinical sign (e.g., bilious vomiting or hematemesis, abdominal distention, gross, or occult blood in the stool) and more than one X-ray finding (e.g., pneumatosis intestinalis, hepatobiliary gas, pneumoperitoneum) (19). Since all eight centers were level III NICU, NEC, and PDA in need of surgery were transferred to other hospitals, resulting in uncompleted data and exclusion from the final analysis. Duration of positive pressure ventilation, days on supplemental oxygen, length of NICU stay, and body weight on discharge were compared.

Short-term safety variable analyses included the following events: transient bradycardia (heart rate <100/min), SpO_2_ < 85%, choking and coughing, laryngeal spasms, or failure of surfactant administration. Investigators were advised to report any events and duration, including the nadir and the way of remission. Data on serious adverse events were collected.

### Statistical Analysis

Based on an estimated incidence of BPD of 25.0% among infants <32 weeks of gestation (20), we calculated an alpha of 0.05 and a power of 90%; 130 infants were enrolled in each group (with a 1:1 design) to detect an absolute difference of 10 percentage points in the incidence of BPD at 36 weeks of corrected age. Therefore, we planned to recruit at least 140 infants in each group, to account for dropouts; 298 patients were enrolled until December 2018.

Analyses were performed on a pre-protocol basis according to a prespecified statistical analysis plan. The incidence of RDS, BPD, PDA, IVH, NEC, and ROP was compared between the two groups. In planned subgroup analyses (51 infants total), smaller infants with gestational age at 25+0 to 29+6 weeks were compared, and assigned to the MISA group and the EISA group, respectively.

Data are expressed as a proportion or mean ± standard deviation (mean ± SD). Proportions were compared by chi-square test or Fisher's exact test analysis. Continuous variables were compared by Student's *t*-test. A two-sided *p* < 0.05 was considered statistically significant. Statistical analysis was performed using IBM SPSS Statistics for Windows, version 22.0 (IBM Corp., Armonk, N.Y., USA).

## Results

### Subjects Characteristics

**Figure 2:**
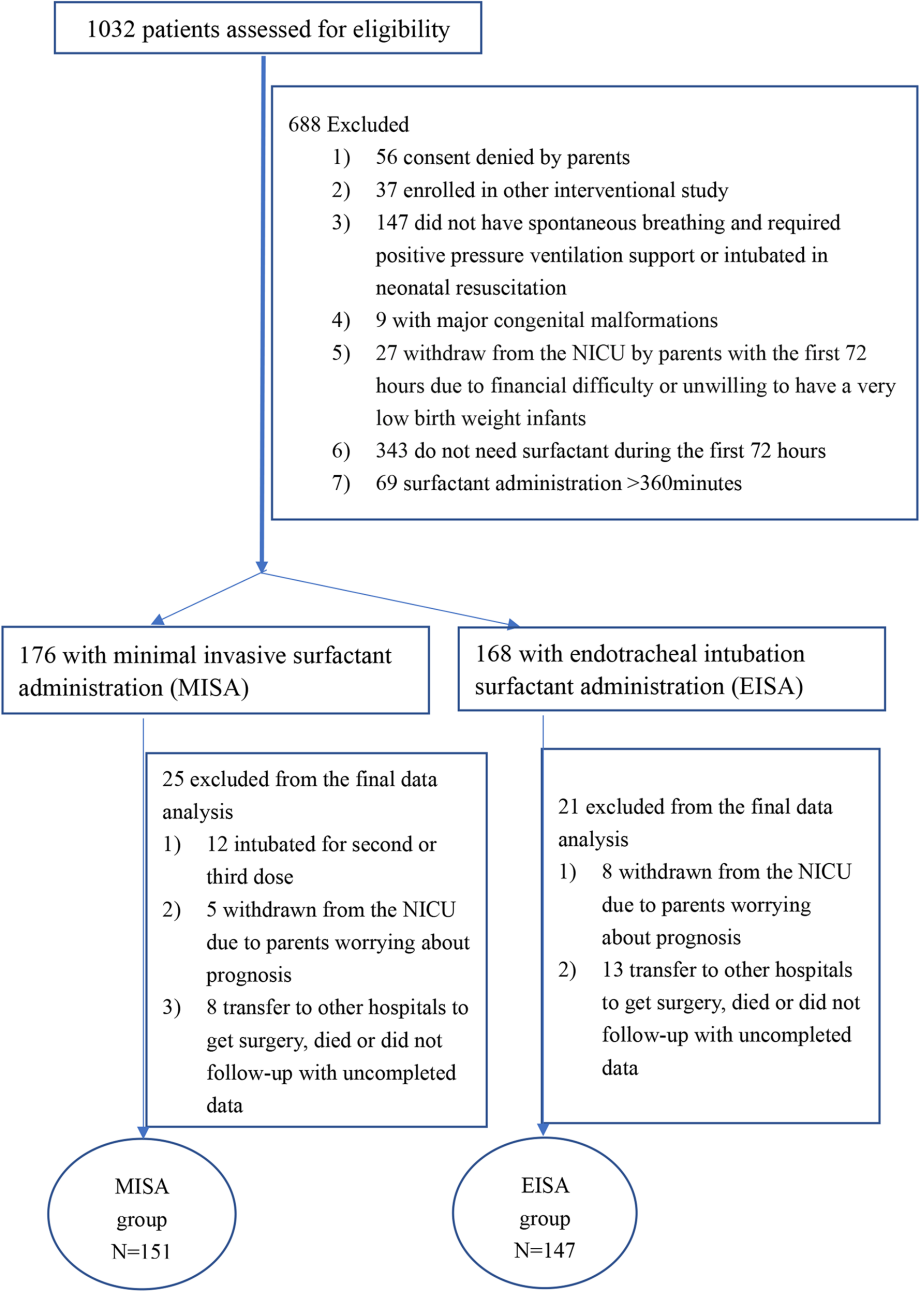
Flowchart of the study population.

During the study period, 1,032 eligible participants were screened at the eight NICUs. However, 688 were excluded due to ≥1 exclusion criterion, or because they did not need surfactant or the surfactant was administrated after 6 h (Figure 2). The study was extended from 12 to 18 months because of low recruitment in some centers. Recruitment rates ranged from 12.8 to 56.5%. Parental consent was obtained within 2 h after admission to NICU if it was not obtained antenatally. A total of 344 infants were enrolled, 176 infants in the MISA group, and 168 infants in the EISA group. In the MISA group, data of 12 patients were excluded from the final data analysis because they were intubated for the second or third dose or intubated ventilation support during the first 72 h after birth. Furthermore, 34 patients in two groups were excluded from the final data analysis due to incomplete data, because they were withdrawn from the NICU care, transferred to other hospitals for surgery, or died before 28 days. Finally, data from 151 infants were analyzed in the MISA group and data from 147 infants were analyzed in the EISA group. The last follow-up was on March 30, 2019.

All preterm infants were treated in the hospitals in which they were born. Mean gestational age and birth weight of the study population was 30.68 ± 1.47 weeks and 1423.22 ± 281.39 g, respectively. Male:female ratio was 165:133. The basal demographic characteristics of subjects are compared in Table 1. The infants receiving MISA were well matched with the infants in the EISA group with similar baseline demographic and clinical characteristics.

**Table 1:** Demographic and clinical characteristics.

**Groups**	**MISA (*n* = 151)**	**EISA (*n* = 147)**	**χ^2^/*t***	***p***
Male*	80 (53.0)	85 (57.8)		0.400
IVF*	19 (12.6)	20 (13.6)		0.794
Antenatal corticosteroids*				0.356
No use	41 (27.1)	33 (22.4)		
Incomplete course	30 (19.9)	24 (16.3)		
Full course	80 (53.0)	90 (61.2)		
Multiple births*	33 (21.9)	38 (25.9)		0.418
Maternal complications*				
Gestational hypertensive diseases	37 (24.5)	36 (24.5)		0.998
Gestational diabetes	21 (13.9)	33 (22.4)		0.056
Premature rupture of membranes	49 (32.5)	43 (29.3)		0.550
Cesarean section*	101 (66.9)	112 (76.2)		0.075
Gestational age, weeks**	30.6 ± 1.6	30.8 ± 1.3	1.298	0.195
Birth weight, g**	1427.6 ± 290.2	1418.7 ± 273.0	0.271	0.786
Birth length, cm**	40.27 ± 3.0	40.1 ± 3.3	0.342	0.733
Birth weight <1000 g*	11 (7.3)	7 (4.8)		0.361
Apgar score**				
1 min	8.6 ± 1.6	8.5 ± 1.8	0.062	0.950
5 min	9.3 ± 1.3	9.2 ± 1.5	0.437	0.662
10 min	9.5 ± 0.7	9.5 ± 0.8	0.640	0.645
Cord blood pH**	7.20 ± 0.06	7.22 ± 0.06	0.550	0.079

Data are expressed as the mean ± SD or number (%). Comparisons were performed with ^*^Pearson χ^2^ or ^**^Student's t test. MISA, minimally invasive surfactant administration; EISA, endotracheal intubation surfactant administration; IVF, in vitro fertilization.

### Primary Outcomes and Complications

Following discharge from the NICU, the proportions of infants with each of the specified primary outcomes were compared between the two groups (Table 2). These outcomes included RDS, requiring two doses of surfactant, pulmonary hemorrhage, BPD, PDA, IVH (grades 1–2 and grades 3–4), white matter injury, pneumonia, clinical sepsis (early or late onset), stage 1 or 2 NEC, and less than stage 3 retinopathy of prematurity. Although the comparison showed no clear benefits of MISA therapy on the incidence of BPD, there was a tendency for a reduction of incidence of BPD (29/151 vs. 138/147, 19.2 vs. 25.9%, *p* = 0.170). As to the incidence of PDA, infants in the EISA group had higher rates compared to infants in the MISA group (60.5 vs. 41.1%, *p* = 0.001).

**Table 2:** Comparison of primary outcomes and complications between two groups.

	**MISA (*n* = 151)**	**EISA (*n* = 147)**	***p***
RDS	139 (92.1)	141 (95.9)	0.161
Requiring two doses of surfactant	18 (11.9)	10 (6.8)	0.130
Pulmonary hemorrhage	2 (1.3)	2 (1.4)	0.679
BPD	29 (19.2)	38 (25.9)	0.170
IVH			0.351
Grades 3–4	10 (6.6)	10 (6.8)	
Grades 1–2	37 (24.5)	26 (17.7)	
White matter injury	7 (4.6)	4 (2.7)	0.381
PDA	62 (41.1)	89 (60.5)	0.001
Pneumonia	63 (41.7)	62 (42.2)	0.937
Neonatal sepsis	24 (15.9)	27 (18.4)	0.481
NEC (stage 1 or 2)	4 (2.6)	2 (1.4)	0.429
ROP	8 (5.3)	11 (7.5)	0.440

*Data are expressed as number (%). Comparisons were performed with Pearson χ^2^. MISA, minimally invasive surfactant administration; EISA, endotracheal intubation surfactant administration; RDS, respiratory distress syndrome; BPD, bronchopulmonary dysplasia; IVH, intraventricular hemorrhages; PDA, patent ductus arteriosus, ROP, retinopathy of prematurity; NEC, necrotizing enterocolitis*.

### Surfactant Administration

Short-term safety variables were evaluated. In the MISA group, tube position was successfully performed in all infants, for 116 (73.62%) infants at the first attempt, and 35 (23.2%) at the second attempt. Transient bradycardia was reported in 17 infants (11.2%) from the MISA group and 12 infants (8.2%) from the EISA group, without differences between the two groups (*p* = 0.367). There was no reported case of occurrence of bloody sputum, apnea, reflux of surfactant, or the need to terminate dosing during surfactant administration.

The surfactant administration was compared between the two groups, including the surfactant infusion time after birth, dose of surfactant, and the qualification of the clinician to practice the surfactant administration (Table 3). The surfactant administration process is shown in Figure 1. The duration of surfactant infusion (Duration 2) in the MISA group (102.16 ± 158.24 s) was significantly longer than that in EISA (43.92 ± 43.29 s) (*p* < 0.0001). The total time of administration of surfactant (Duration 3) was significantly different between the two groups (122.24 ± 163.74 vs. 65.16 ± 73.84, *t* = 3.896, *p* < 0.0001). Although the clinicians' working experience between the two groups was not significant, the clinicians who practiced minimally invasive therapy had ~1 more year of experience compared to those who practiced intubation therapy.

**Table 3:** Comparison of surfactant administration between two groups.

	**MISA (*n* = 151)**	**EISA (*n* = 147)**	***t***	***p***
Surfactant administration time after birth, h	2.5 ± 1.7	2.47 ± 1.7	0.716	0.475
Dose of surfactant, mg/kg	100.0 ± 14.3	101.5 ± 24.9	0.645	0.519
Duration 1, s	20.5 ± 45.4	15.4 ± 15.5	1.289	0.198
Duration 2, s	102.2 ± 158.2	43.9 ± 43.3	4.358	0.000
Duration 3, s	122.2 ± 163.7	65.2 ± 73.8	3.896	0.000
Working experiences in pediatrics, years	8.5 ± 5.4	7.7 ± 5.2	1.377	0.169
Working experiences in neonatology, years	7.3 ± 4.5	6.5 ± 4.6	1.553	0.122
Working experiences in NICU, years	6.5 ± 4.4	5.8 ± 4.5	1.267	0.206

*Data are expressed as mean ± SD. Comparisons were performed with Student's t test. Comparisons were performed with Pearson χ^2^. MISA, minimally invasive surfactant administration; EISA, endotracheal intubation surfactant administration; Duration 1, placing catheter time, seconds; Duration 2, surfactant infusion duration through the catheter, seconds; Duration 3, total time of administration of surfactant, from introducing of laryngoscope by mouth, placing catheter, surfactant infusion to withdrawn of the catheter, seconds*.

### Blood gas Analysis

The blood gas analysis of two groups of infants was done at three time points, before surfactant and 1 h and 12 h post surfactant administration (Table 4). The arterial pH, the partial pressure of arterial oxygen (PaO_2_), partial pressure of arterial carbon dioxide (PaCO_2_), and the arterial oxygen saturation (SaO_2_) value were not significantly different between the two groups at three time points.

**Table 4:** Blood gas comparison between two groups at three-time points.

		**MISA (*n* = 151)**	**EISA (*n* = 147)**	***t***	***p***
**Pre-surfactant**					
	PH	7.30 ± 0.18	7.29 ± 0.09	0.401	0.682
	PO_2_, mmHg	80.15 ± 28.28	77.80 ± 25.87	0.743	0.458
	PCO_2_, mmHg	47.56 ± 14.21	46.26 ± 12.37	0.830	0.407
	SaO_2_, %	93.60 ± 6.75	93.68 ± 5.17	0.115	0.908
	BE, mmol/L	−5.04 ± 2.82	−4.50 ± 2.76	1.651	0.100
**Post-surfactant**					
1 h	PH	7.35 ± 0.09	7.34 ± 0.09	0.524	0.600
	PO_2_, mmHg	75.72 ± 28.56	73.68 ± 20.89	0.685	0.494
	PCO_2_, mmHg	37.20 ± 10.85	38.89 ± 12.30	1.212	0.227
	SaO_2_, %	93.97 ± 6.18	94.38 ± 6.75	0.510	0.611
	BE, mmol/L	−4.53 ± 2.59	−4.15 ± 2.97	1.184	0.237
12 h	PH	7.34 ± 0.09	7.35 ± 0.10	1.103	0.271
	PO_2_, mmHg	76.29 ± 23.15	76.54 ± 22.91	0.094	0.925
	PCO_2_, mmHg	38.16 ± 9.38	38.39 ± 9.15	0.222	0.825
	SaO_2_, %	93.85 ± 5.24	94.79 ± 5.61	1.497	0.135
	BE, mmol/L	−3.81 ± 2.67	−3.37 ± 2.67	1.411	0.159

*Data are expressed as mean ± SD. Comparisons were performed with Student's t test. PO_2_, partial pressure of oxygen; PCO_2_, the partial pressure of carbon dioxide; SaO_2_, arterial saturation of peripheral oxygen; BE, base excess*.

### Heart Rate and Blood Pressure

Heart rate, systolic blood pressure, and diastolic blood pressure were compared between the two groups before surfactant administration and 1, 6, 12, and 24 h after surfactant administration (Table 5). A slightly increased heart rate was noted 1 h post surfactant administration in the EISA group ([139.63 ± 10.54] bpm) compared to the MISA group ([136.72 ± 13.36] bpm, *t* = 2.086, *p* = 0.038); the observed difference was statistically significant.

**Table 5:** Comparison of heart rate and blood pressure between two groups.

		**MISA (*n* = 151)**	**EISA (*n* = 147)**	***t***	***p***
**Pre-surfactant**					
	HR (bpm)	137.6 ± 11.1	136.3 ± 14.9	0.829	0.408
	SBP (mmHg)	61.0 ± 8.1	59.8 ± 9.6	1.247	0.213
	DBP (mmHg)	32.4 ± 6.1	32.3 ± 7.6	0.158	0.875
**Post-surfactant**					
1 h	HR_1_ (bpm)	136.7 ± 13.4	139.6 ± 10.5	2.086	0.038
	SBP_1_ (mmHg)	60.9 ± 8.5	59.5 ± 8.0	1.488	0.138
	DBP_1_ (mmHg)	32.8 ± 7.3	33.2 ± 6.3	0.545	0.586
6 h	HR_6_ (bpm)	137.3 ± 10.8	139.1 ± 10.5	1.419	0.157
	SBP_6_ (mmHg)	61.7 ± 7.2	61.4 ± 7.1	0.428	0.669
	DBP_6_ (mmHg)	32.7 ± 5.4	33.0 ± 5.0	0.491	0.624
12 h	HR_12_ (bpm)	136.4 ± 11.4	136.7 ± 11.3	0.292	0.770
	SBP_12_ (mmHg)	62.1 ± 7.6	61.1 ± 6.8	1.134	0.258
	DBP_12_ (mmHg)	33.0 ± 6.3	32.9 ± 4.8	0.294	0.769
24 h	HR_24_ (bpm)	137.0 ± 11.1	137.1 ± 10.0	0.098	0.922
	SBP_24_ (mmHg)	62.9 ± 8.3	62.8 ± 7.9	0.113	0.910
	DBP_24_ (mmHg)	34.1 ± 5.4	34.0 ± 5.2	0.085	0.932

*Data are expressed as mean ± SD. Comparisons were performed with Student's t test. MISA, minimally invasive surfactant administration; EISA, endotracheal intubation surfactant administration; HR heart rate (bpm); SBP, systolic blood pressure (mmHg); DB, diastolic blood pressure (mmHg)*.

### Respiratory Support and Discharging Data

There were no differences in the duration of nCPAP respiratory support and supplemental oxygen (Table 6) between the two groups. Infants in both groups stayed at NICU for nearly 40 days, with around 36 weeks corrected gestational age and gained similar body weight.

**Table 6:** Comparison of respiratory support and discharge data between two groups.

	**MISA (*n* = 151)**	**EISA (*n* = 147)**	***t***	***p***
nCPAP duration, days	10.3 ± 12.2	10.7 ± 11.2	0.286	0.755
Supplemental oxygen duration, days	15.7 ± 16.4	16.1 ± 15.7	0.237	0.813
Hospitalization days, days	39.8 ± 17.1	39.5 ± 15.4	0.201	0.841
Corrected gestational age, weeks	36.1 ± 2.1	36.3 ± 1.8	1.098	0.273
Body weight, g	2075.2 ± 217.1	2071.5 ± 191.3	0.157	0.875

*Data are expressed as mean ± SD. Comparisons were performed with Student's t test. MISA, minimally invasive surfactant administration; EISA, endotracheal intubation surfactant administration; nCPAP, nasal continuous positive airway pressure*.

### Subgroup Analysis

In the subgroup analysis, demographic, clinical characteristics, and neonatal complications of 51 smaller preterm infants (defined as 25+0 weeks to 29+6 weeks) between the two groups, named minimally invasive administration smaller (MISAs) group and endotracheal intubation administration smaller (EISAs) group, were compared (Table 7). The comparison showed a significant reduction in morbidity of BPD (9/31, 29.0 vs. 14/20, 70.0%, *p* = 0.004) and PDA (9/31, 29.0, vs. 13/21, 65.0%, *p* = 0.011).

**Table 7:** A subgroup comparison of smaller preterm infants between two groups.

**Groups**	**MISAs (*n* = 31)**	**EISAs (*n* = 20)**	***t***	***p***
Gestational age, weeks**	28.1 ± 1.2	28.4 ± 0.5	0.471	0.195
Birth weight, g**	1140.8 ± 187.6	1167.5 ± 203.7	0.271	0.786
RDS*	27 (87.1)	20 (100.0)		0.094
BPD*	9 (29.0)	14 (70.0)		0.004
IVH*				0.176
Grades 3–4	3 (9.7)	0 (0)		
Grades 1–2	10 (32.3)	4 (20.0)		
White matter injury*	2 (6.5)	1 (5.0)		0.830
PDA*	9 (29.0)	13 (65.0)		0.011
Pneumonia*	13 (41.9)	7 (35.0)		0.771
Clinical sepsis (early or late onset) *	7 (22.6)	5 (25.0)		0.842
ROP*	4 (12.9)	5 (25.0)		0.269

Data are expressed as the mean ± SD or number (%). Comparisons were performed with ^*^Pearson χ^2^ or ^**^Student's t test. MISA, minimally invasive surfactant administration; EISA, endotracheal intubation surfactant administration; RDS, respiratory distress syndrome; BPD, bronchopulmonary dysplasia; IVH, intraventricular hemorrhages; PDA, patent ductus arteriosus, ROP, retinopathy of prematurity; NEC, necrotizing enterocolitis.

The blood gas analysis of small preterm infants was compared at two time points, 1 and 12 h post surfactant administration (Table 8). Arterial oxygen saturation (SaO_2_) value was higher in the EISAs group (96.06 ± 4.10) compared with the MISAs group (91.68 ± 8.31) at 1 h (*t* = 2.075, *p* = 0.044). In the EISAs group, at 12 h after surfactant administration, the partial pressure of arterial oxygen (PaO_2_, [82.33 ± 23.24] vs. [67.46 ± 19.54], *t* = 2.461, *p* = 0.017) and SaO_2_ ([96.39 ± 3.23] vs. [92.67 ± 6.32], *t* = 2.422, *p* = 0.019) were higher than in the MISAs group.

**Table 8:** A subgroup post-surfactant blood gas comparison of smaller preterm infants between two groups.

		**MISAs (*n* = 31)**	**EISAs (*n* = 20)**	***t***	***P***
1 h	PH	7.35 ± 0.10	7.33 ± 0.75	0.806	0.424
	PO_2_, mmHg	69.31 ± 20.89	78.97 ± 24.97	0.344	0.156
	PCO_2_, mmHg	38.67 ± 12.12	34.27 ± 9.59	1.310	0.197
	SaO_2_, %	91.68 ± 8.31	96.06 ± 4.10	2.075	0.044
	BE, mmol/L	−3.85 ± 2.79	−4.86 ± 1.80	1.371	0.177
12 h	PH	7.33 ± 0.09	7.30 ± 0.12	1.200	0.236
	PO_2_, mmHg	67.46 ± 19.54	82.33 ± 23.24	2.461	0.017
	PCO_2_, mmHg	36.82 ± 9.58	39.95 ± 8.74	1.181	0.243
	SaO_2_, %	92.67 ± 6.32	96.39 ± 3.23	2.422	0.019
	BE, mmol/L	−3.88 ± 3.20	−4.37 ± 2.13	0.608	0.546

*Data are expressed as mean ± SD. Comparisons were performed with Student's t test. PO_2_, partial pressure of oxygen; PCO_2_, the partial pressure of carbon dioxide; SaO_2_, arterial saturation of peripheral oxygen; BE, base excess*.

## Discussion

In this study, we evaluated whether MISA was applicable in infants at 25 weeks to 31 + 6 weeks gestational age in China, and whether this method of surfactant administration decreases incidence of BPD at 36 weeks corrected gestational age compared with intubation surfactant administration therapy. Although the comparison showed no clear benefits of MISA therapy on the reduction of BPD in infants less than 32 weeks gestational age, a reduced incidence of BPD was observed in smaller infants, i.e., less than 30 weeks gestational age. A significant reduction rate of survival without PDA in the MISA group was demonstrated. The duration of required MISA was as long as 5 min, which was longer than the intubation therapy. Fluctuations in heart rate of intubated infants were observed more than MISA infants. Subgroup comparison of smaller infants (<30 weeks) in the minimally invasive group showed a significant reduction in morbidity of BPD and PDA. Also, according to blood gas analysis, SaO_2_ and PaO_2_ values of smaller infants were relatively lower compared with intubation controls.

Over the last decade, there have been many changes in the ventilation support and the modality of exogenous surfactant administration to RDS infants (21). Due to the potential injury of intubation and positive pressure ventilation to the immature lung, attempts have been made to use non-invasive methods in the management of these patients. The nCPAP has been recommended as the initial respiratory support for spontaneous breathing infants (6). However, in the COIN study, more than 50% of ELBWs failed on nCPAP (22), although stabilization in the delivery room with nCPAP was successful. LISA and MIST reflect the efforts of neonatologists for pursuing the most protective technique to the most immature lung of the ELBW infants (10–12, 23). The core of these less invasive techniques involves the use of a thin catheter (24).

In the current study, we named our method MISA for the following three reasons. First, there is a potential for injury to the mucosa of upper airway because of the introduction of direct laryngoscope, so we used the word “minimally.”A 5F end hole gastric tube catheter and ophthalmic forceps were used through the mouth into the pharynx directly under laryngoscopy. Second, since Magill forceps were unavailable in our NICUs, the small ophthalmic forceps were used as an alternative to grasp the gastric tube catheter, which was made of soft polyethylene. This technique may be easier for those who are not experienced in Magill forceps. Third, we kept the infant's mouth closed and continued nCPAP during surfactant administration. Our method had more or less differences with the LISA and the MIST, so we named it MISA.

In the current study, MISA was successfully performed in all eight NICUs, although in some cases, a second attempt was needed. However, in this study, the clinicians that practiced minimally invasive therapy had approximately one more year of experience compared to the ones who practiced intubation therapy, which means that clinicians need one additional year of training in the neonatal endotracheal intubation.

In this study, the surfactant was instilled by hand as long as 300 s by mini-boluses. Cases requiring intubation and positive pressure ventilation support during the first 72 h in the MISA group were excluded because even a brief exposure to positive pressure ventilation has been shown to be related to the occurrence of BPD and to induce ventilator-associated lung injury (25). Although potential adverse events were previously reported, only bradycardia was detected in this study. In cases with bradycardia, surfactant infusion was suspended until heart rates recovered to normal while nCPAP was continued (26). Also, there was a slightly increased heart rate in intubation therapy after 1 h of surfactant administration (27). Consequently, we believe that MISA is applicable to the level III NICUs in China, provided there is a staff experienced in neonatal endotracheal intubation.

BPD affects more than 30% of infants with body weight <1,250 g at birth. Despite the use of surfactant and NICU advances permitting more premature infants to survive, the incidence of BPD is slowly growing (7). The less invasive method of surfactant injection has attracted more attention in the neonatal respiratory diseases sector. In a meta-analysis, Lau et al. compared subjects in the thin catheter group with the INSURE group. They found that fewer infants developed BPD (18/166 [10.8%] vs. 27/162 [16.7%]). Forest plot showed a 34.4% reduction in the risk of BPD (RR = 0.656; 95% CI = 0.375–1.149; *p* = 0.141) without statistical significance (28). Several previous studies enrolled subjects with younger gestational age and birth weight compared to our MISA study. In the Take Care study, Kanmaz et al. recruited 100 RDS infants in the LISA group with 28.3 ± 2 gestational age and mean birth weight 1093 ± 270 g (29). The subjects in the study of Mohammadizadeh et al. had a gestational age of 30 ± 2 weeks and a birth weight of 1289 ± 219 g (30). Another study in China conducted by Bao et al. enrolled infants in LISA with a gestational age of 29.1 ± 1.5 weeks and a birth weight of 1034 ± 221 g (27). In these three mentioned studies, researchers proved that the BPD rate was significantly lower in the thin catheter group (29).

There was no apparent benefit of MISA therapy on the incidence of BPD in this study. An alternative explanation for the unfavorable outcome for this study population was the relatively larger gestational age and birth weight, which was a limitation of the study. We speculate that reason for this finding may be the strategy of initial respiratory support and the optimal time of surfactant supplement. As we know, the CPAP stabilizes the alveoli, lessens retraction during inspiration, improves compliance, and respiratory mechanics. This leads to a better gas exchange. In NRDS infants, surfactant supplement at the optimal time decreases surface tension, leading to increased compliance and improved functional residual capacity. In smaller NRDS infants, the action between CPAP support and surfactant supplement may have better effectiveness. Then, in the subgroup analysis, there is a significant reduction rate of survival without BPD in infants <30 weeks gestational age in the MISAs group. Although it seems to suggest a particular patient subgroup where the technique could be more useful, the cases with intubation and positive pressure ventilation support during the first 72 h were excluded in the data analysis in the current study. Thus, we could only prove that the incidence of BPD was not different between the two groups. It cannot be decided if it were minimally invasive therapy per se, never being intubated, or never being under invasive ventilation support that reduced BPD in infants smaller than 30 weeks gestational age.

A significant reduction rate of survival without PDA was demonstrated in minimally invasive therapy, which is the most promising result of the study. Pulmonary circulation embryology is intrinsically related to cardiovascular and pulmonary development. The transition from intrauterine to extrauterine life is a critical phase in physiological adaptation, which impacts many organ systems, especially the heart and the lungs (31). While alterations in the respiratory system can significantly affect cardiovascular function, the opposite is also exact. Infants with RDS had decreased lung compliance and increased airway resistance, which led to increased work of breathing, alveolar hypoventilation, and CO_2_ retention. In more severe cases, this can also compromise oxygenation and cardiac function. The deleterious effects of the PDA with significant left to right shunting and increased pulmonary blood flow on lung function are also well established (32). A hemodynamically relevant PDA has been associated with pulmonary edema and respiratory compromise has been associated with a resultant higher incidence of BPD. Excessive pulmonary blood flow via the pulmonary vascular bed results in an interrupted lung structure similar to the characteristic BPD changes in immature infants with extremely low birth weight (33). Therefore, a more stable hemodynamics with less PDA could explain the significant reduction in morbidity of BPD in the MISAs group in the subgroup analysis.

Intubation resulted in higher levels of arterial oxygen saturation (SaO_2_) values at 1 h and higher levels of SaO_2_ and PaO_2_ values at 12 h after surfactant administration in the subgroup comparison of smaller infants. Although the mechanisms of BPD development were complicated, hyperoxia damage was thought to be one of the main factors (34). Although necessary to sustain the preterm infant's life, relatively high oxygenation, and positive pressure ventilation damage the lung by dysregulation of the growth of the pulmonary vasculature and the lung parenchyma. It has been established that intubation and exposure to high concentrations of oxygen, even for a brief period, may cause harm, predisposing infants to lung injury and subsequent BPD (25). In a meta-analysis, Askie et al. collected the data from five clinical trials completed between 2005 and 2014 (35). A total of 4,965 infants born before 28 weeks' gestation were randomized to two SpO_2_ target range groups, the lower (85–89%) and the higher (91–95%). The results suggested that the lower group was related to a higher risk of death and NEC, and a lower risk of ROP and BPD. Therefore, lower levels of arterial oxygen saturation (SaO_2_) values at 1 h and lower levels of SaO_2_ and PaO_2_ values at 12 h could explain the significant reduction in morbidity of BPD in the MISAs group in the subgroup analysis.

There are some limitations to this study. First, as mentioned above, the subjects had relatively larger gestational age and birth weight. Second, group allocation was not blinded, so it is possible that the clinical treatment of the individual infant was influenced by the treating clinicians knowing the group assignment. Third, although extubation criteria were established, there were eight NICUs in the study, which means that the extension of positive pressure ventilation in some cases of the intubation group could not be ruled out, thus resulting in longer days of positive pressure ventilation and an ultimately higher rate of BPD. Future study in this field is required to consider the abovementioned factors, i.e., surfactant administration by minimally invasive or INSURE, intubated or never intubated, and invasive or non-invasive ventilation support.

## Conclusion

MISA was not superior in relation to the primary outcome of the study, but it was related to benefits in reducing the incidence of PDA, which suggests less hemodynamic interference in infants with extremely/very low birth weight during the critical transition phase of physiological adaptation shortly after birth. MISA is an applicable therapy for extremely/very low birth weight preterm infants with respiratory distress syndrome. However, before its endorsement in all regions, clinicians should undergo necessary training to gain more experience.

## What Is Known

Minimally invasive surfactant administration to the spontaneous breathing preterm infants with nasal continuous positive airway pressure support could reduce the risk of bronchopulmonary dysplasia.

## What Is New

Minimally invasive surfactant administration is associated with a decreased incidence of patent ductus arteriosus in infants with extremely/very low birth weight.

## Data Availability Statement

The datasets generated for this study are available on request to the corresponding author.

## Ethics Statement

The studies involving human participants were reviewed and approved by Peking University Third Hospital Medical Science Research Ethics Committee (M2017160). Written informed consent to participate in this study was provided by the participants' legal guardian/next of kin.

## Author Contributions

TH, HL, HZ, XuZ, FS, XiZ, MZ, LB, WL, RJ, XTi, QG, WZ, and LL made contributions to the conception or design of the work or data acquisition, analysis, and interpretation. TH, HL, HZ, and XTo performed formal analysis and data curation, drafted the work, or critically revised for important intellectual content. TH, MG, YD, XL, FL, MX, JZ, WG, and XTo provided approval for publication of the content. All authors agree to be responsible for all aspects of the work to ensure proper investigation and resolution of issues related to the accuracy or integrity of any part of the work.

## Conflict of Interest

The authors declare that the research was conducted in the absence of any commercial or financial relationships that could be construed as a potential conflict of interest.
